# Remdesivir and Its Combination With Repurposed Drugs as COVID-19 Therapeutics

**DOI:** 10.3389/fimmu.2022.830990

**Published:** 2022-05-12

**Authors:** Bhaswati Chatterjee, Suman S. Thakur

**Affiliations:** ^1^ Chemical Science, National Institute of Pharmaceutical Education and Research, Hyderabad, India; ^2^ Proteomics and Cell Signaling, Centre for Cellular and Molecular Biology, Hyderabad, India

**Keywords:** repurposed drugs, therapeutics, COVID-19, remdesivir, baricitinib

## Abstract

The SARS-CoV-2 virus needs multiple copies for its multiplication using an enzyme RNA-dependent RNA polymerase (RdRp). Remdesivir inhibits viral RdRp, controls the multiplication of the virus, and protects patients. However, treatment of COVID-19 with remdesivir involves adverse effects. Many ongoing clinical trials are exploring the potential of the combination of remdesivir with repurposed drugs by targeting multiple targets of virus and host human simultaneously. Better results were obtained with the remdesivir–baricitinib combination treatment for COVID-19 compared to the treatment with remdesivir alone. Notably, recovery from COVID-19 was found to be 8 days less *via* the remdesivir–baricitinib combination treatment as compared to remdesivir treatment alone. Furthermore, the mortality rate *via* the remdesivir–baricitinib combination treatment was lower compared to the remdesivir-only treatment. Remdesivir targets the SARS-CoV-2 enzyme while baricitinib targets the host human enzyme. Simultaneously, remdesivir and baricitinib as a combination inhibit their target viral RdRp and human Janus kinase, respectively. Ongoing trials for the combination of drugs will suggest in the future whether they may reduce the recovery time, reduce the mortality rate, and improve patient clinical status for noninvasive ventilation. In the future, simultaneously targeting virus replication enzymes and host human kinases may be the strategy for SARS-CoV-2 therapeutics.

## Introduction

Coronavirus disease 2019 (COVID-19) is a severe infectious viral disease. This disease first emerged in Wuhan, China and was caused by SARS-CoV-2 infection (1). Notably, SARS-CoV-2 belongs to the β-coronavirus genus with lineage B and is a single-stranded RNA virus (2). There is an 89.8% sequence identity in spike (S) proteins of SARS-CoV-2 and SARS-CoV (3). SARS-CoV-2 infects not only humans but also companion animals after their close contact with infected humans (4–6). The transmission of SARS-CoV-2 occurs through body fluids including saliva and through aerosol particles. SARS-CoV-2 damages the respiratory tract system and causes respiratory inconvenience with low oxygen and also develops symptoms related to cardiovascular and neurological diseases (7, 8). Unfortunately, SARS-CoV-2 creates havoc in patients with other comorbidities including compromised immunity, hypertension, diabetes, and respiratory and cardiovascular diseases (9).

There are several potential repurposed drugs for COVID-19. Obatoclax showed antiviral activity *in vitro* against SARS-CoV-2 entry by blocking endocytosis and the membrane fusion pathway (10). Furthermore, obatoclax inhibits the pan-BCL-2 family of proteins, including MCL-1, and imposes anti-influenza A virus (IAV) activity as it inhibits MCL-1 (11, 12). β-d-N4-hydroxycytidine (NHC; EIDD-1931) showed antiviral activity and dose-dependent reduction in virus titers with an IC_50_ of 0.08 µM against SARS-CoV-2 (13). Gysi et al. attempted to find repurposed drugs against SARS-CoV-2 using a network medicine framework and tried to rank 6340 drugs against SARS-CoV-2 using artificial intelligence, network diffusion, and proximity. They experimentally screened 918 drugs in VeroE6 cells. Later, they also screened top-ranked drugs in human cells with a 62% success rate. Finally, they selected six drugs for repurposing against SARS-CoV-2 including azelastine, digoxin, and auranofin (14). Interestingly, low doses of rapamycin were proposed as a therapy for COVID-19 treatment as they control cytokine storms and virus particles (15). Rapamycin inhibits mTORC1 and pro-inflammatory cytokines including IL-2, IL-6, and IL-10, and blocks the G1-to-S phase transition in the cell cycle. Furthermore, mTOR inhibition leads to blockage of downstream activation and inhibition of the virus cell-line overgrowth (16). Rapamycin inhibits the viral protein synthesis of pUL44 and pp65 in Cytomegalovirus (CMV) that are needed for replication in macrophages (17). SARS-CoV-2 interacts with LARP1 and FKBP7 human proteins regulated by the mTORC1 pathway. Furthermore, Chakravarty et al. have suggested that the combinatorial therapy of angiotensin-converting enzyme (ACE) inhibitors and calcium channel blockers (CCBs) will be more beneficial compared to a single-drug approach using AI-integrated mechanistic modeling (18). Anti-diabetic and anti-aging natural compounds may be used for the treatment of COVID-19 as about 35% of COVID-19 patients who died suffered from diabetes (19). Interestingly, 3HP-β-LG was also effective against SARS-CoV-2 in Vero-E6 cells with an IC_50_ of 2.31 μM, and may be repurposed for therapeutics against SARS-CoV-2 infection (20).

The JAK inhibitor baricitinib was used to treat COVID-19 (21–24). Baricitinib with the current standard of care was able to reduce overall death in 28 days and also in 60 days during COVID-19 treatment (25). Furthermore, the safety profile was the same between the placebo and baricitinib treatment groups. Interestingly, baricitinib has multiple targets. It inhibits both endocytosis regulators AP2-associated protein kinase 1 (AAK1) and cyclin G-associated kinase (GAK) for endocytosis. Moreover, to control inflammation, cytokine signaling is inhibited, preventing SARS-CoV-2 infection (26). Additionally, it helps in improving oxygenation, increasing lymphocyte count in COVID-19 patients, and reducing inflammation markers (23, 27–33). Baricitinib also has the potential to reduce mortality in COVID-19 patients in combination with standard care including dexamethasone at baseline.

Furthermore, Janus-associated kinase (JAK) inhibitors including ruxolitinib and tofacitinib were also able to reduce the mortality rate in COVID-19 (34, 35). The phase II trial on patients with severe COVID-19 was carried out using ruxolitinib, a JAK inhibitor plus standard-of-care treatment. The computed tomography improvement at day 14 was shown in 90% of patients having ruxolitinib treatment compared to 61.9% of patients in the control group. No death was observed in the ruxolitinib treatment group on day 28 while three patients died in the control group. Moreover, ruxolitinib was well tolerated with low toxicities and no adverse side effects were reported. Additionally, a significant decrease in the levels of seven cytokines was observed in the ruxolitinib treatment group compared to the control group. Numerically, faster clinical improvement was observed in ruxolitinib when applied as therapeutics against COVID-19. Furthermore, ruxolitinib has the potential to treat SARS-CoV-2 infection as it helped in faster recovery from lymphopenia, with an improvement in chest computed tomography and a favorable side-effect profile in COVID-19 patients (34).

A study found that treatment with tofacitinib showed a lower risk of respiratory failure or death through day 28 compared to placebo in the hospitalized patients with COVID-19 (35). There was an 18.1% cumulative incidence of death or respiratory failure through day 28 in the case of the tofacitinib treatment group compared to 29.0% in the placebo group. Furthermore, 2.8% of patient deaths were observed from any cause through day 28 in the tofacitinib treatment group while 5.5% of death occurred in placebo. There are several ongoing trials related to tofacitinib (ClinicalTrials.gov numbers NCT04415151 and NCT04750317) and baricitinib (NCT04390464, NCT04640168, NCT04421027, and NCT04381936) that will provide more evidence of the success of these JAK inhibitors as therapeutics for COVID-19 (35).

**Figure 1:**
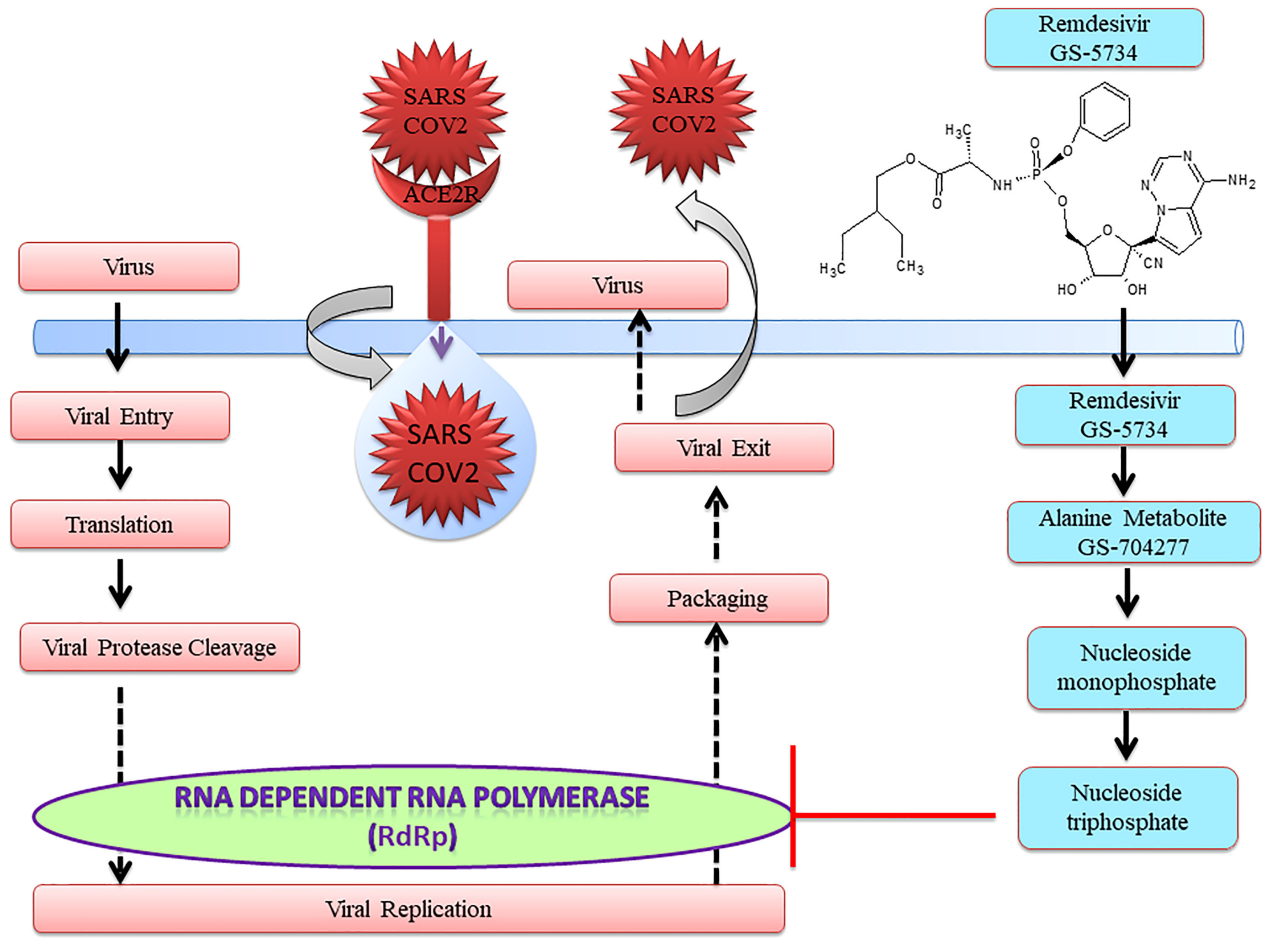
Schematic representation of remdesivir as a repurposed drug for COVID-19.

**Figure 2:**
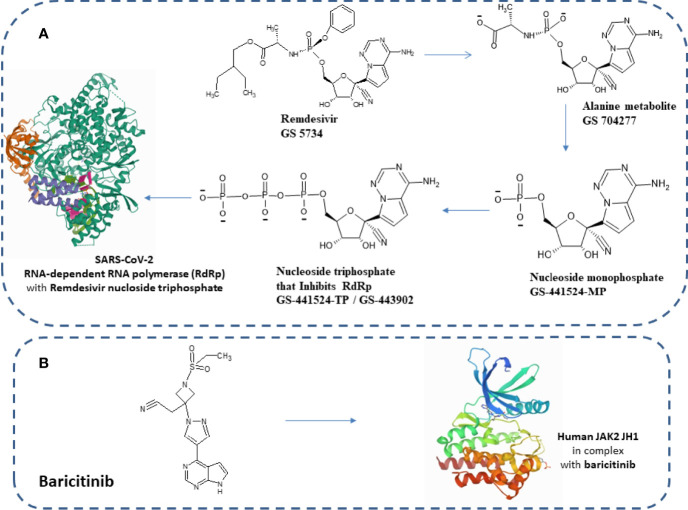
**(A)** Chemical structure of remdesivir and its metabolites. Structure of elongating SARS-CoV-2 RdRp with remdesivir (PDB structure ID: 7B3B). **(B)** Human JAK2 JH1 in combination with baricitinib (PDB structure ID: 6VN8).

Notably, US-FDA has approved remdesivir as an emergency drug for the treatment of COVID-19 on October 22, 2020 (**Figure 1**) on adults and children (36, 37). It is an adenosine analog nucleotide monophosphoramidate pro-drug. Furthermore, it also acts as an antiviral and RNA polymerase inhibitor. After entering the cell, remdesivir (GS 5734) undergoes several changes such as from alanine metabolite (GS 704277) to nucleoside monophosphate and finally to nucleoside triphosphate that inhibits RNA-dependent RNA polymerase (RdRp) (**Figure 2**). Recently, a cryo-electron microscopy (EM)-derived RdRp structure with remdesivir nucleoside triphosphate was obtained at 3.1 Å resolution (38, 39) (**Figure 2**). The cryo-EM structure of SARS-CoV-2-RdRp with remdesivir suggested a partial double-stranded RNA template that enters into the central channel of RdRp (40). Notably, it also reveals that remdesivir terminates chain elongation after covalently binding into the primer strand at the first replicated base pair. The docking result also suggested that remdesivir tightly binds with RdRp of SARS-CoV-2 (41).

During COVID-19 treatment, 5 mg/kg of remdesivir was given on the first day while 2.5 kg/day was given from the second day onwards for children weighing between 3.5 and 40 kg. The dose of remdesivir administered to adults was 200 mg on the first day while 100 mg was administered from the second day onwards. The duration of the treatment depended on the condition of the patient (37).

**Figure 3:**
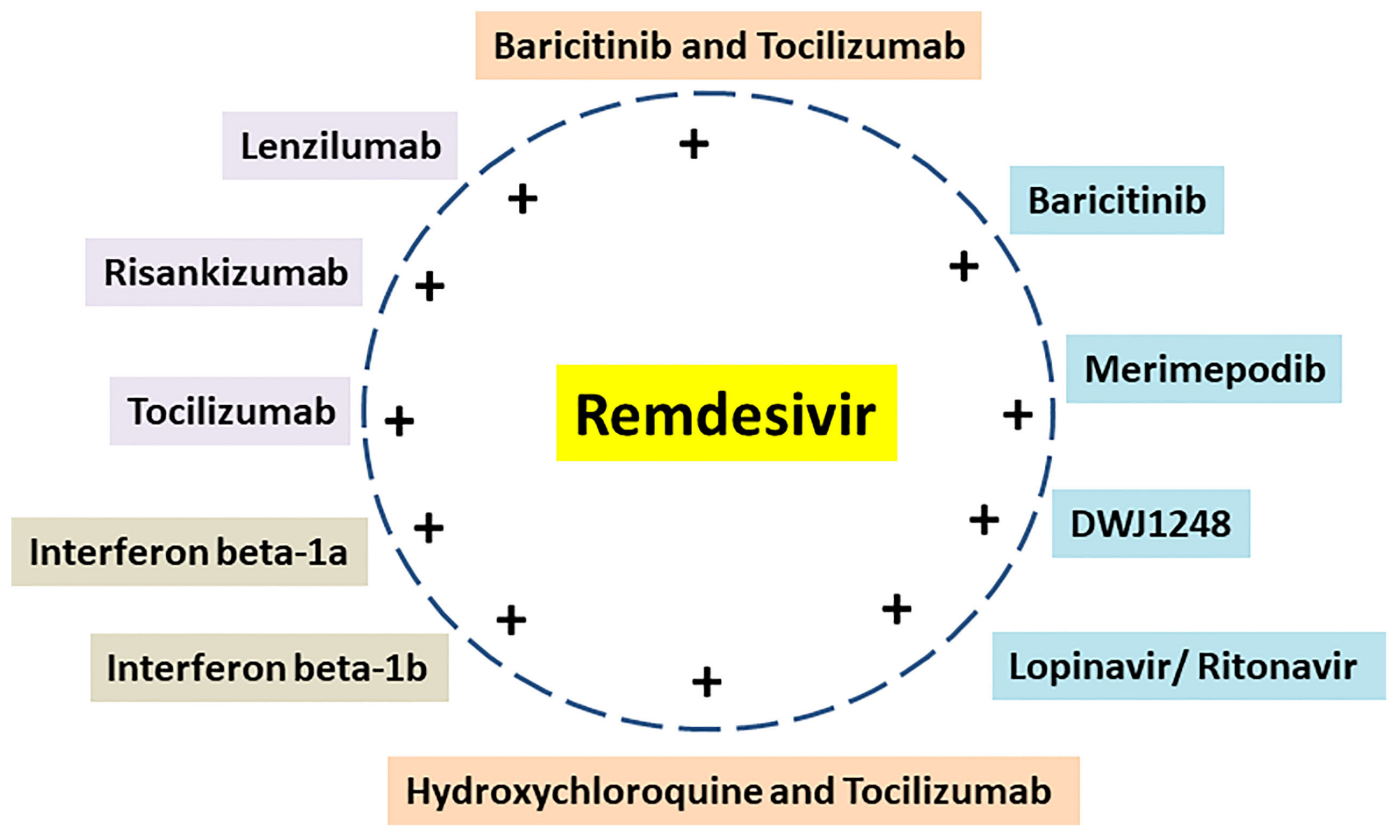
Remdesivir and its combination with repurposed drugs under clinical trial for COVID-19 therapeutics.

**Figure 4:**
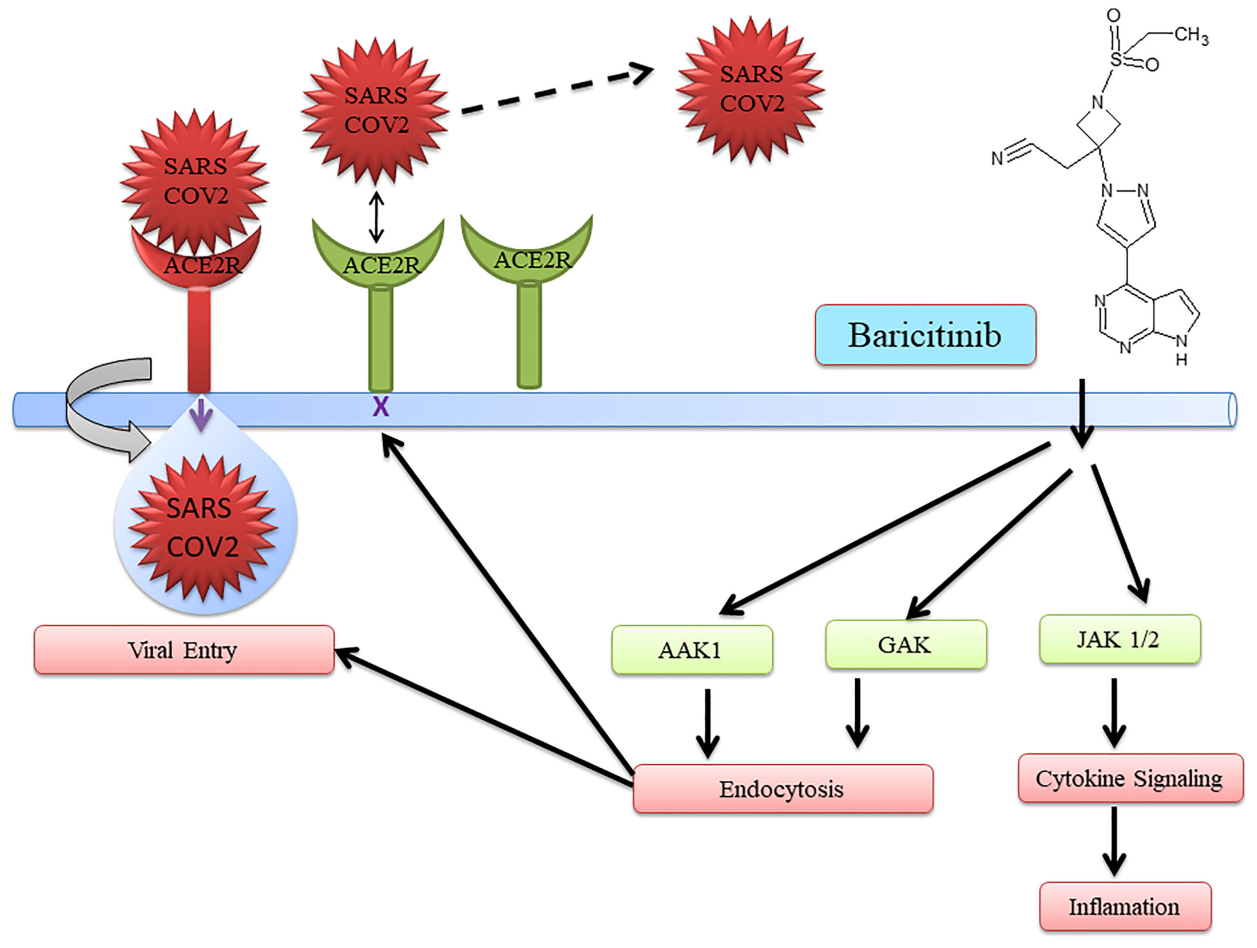
Schematic representation of baricitinib as a repurposed drug for COVID-19.

**Figure 5:**
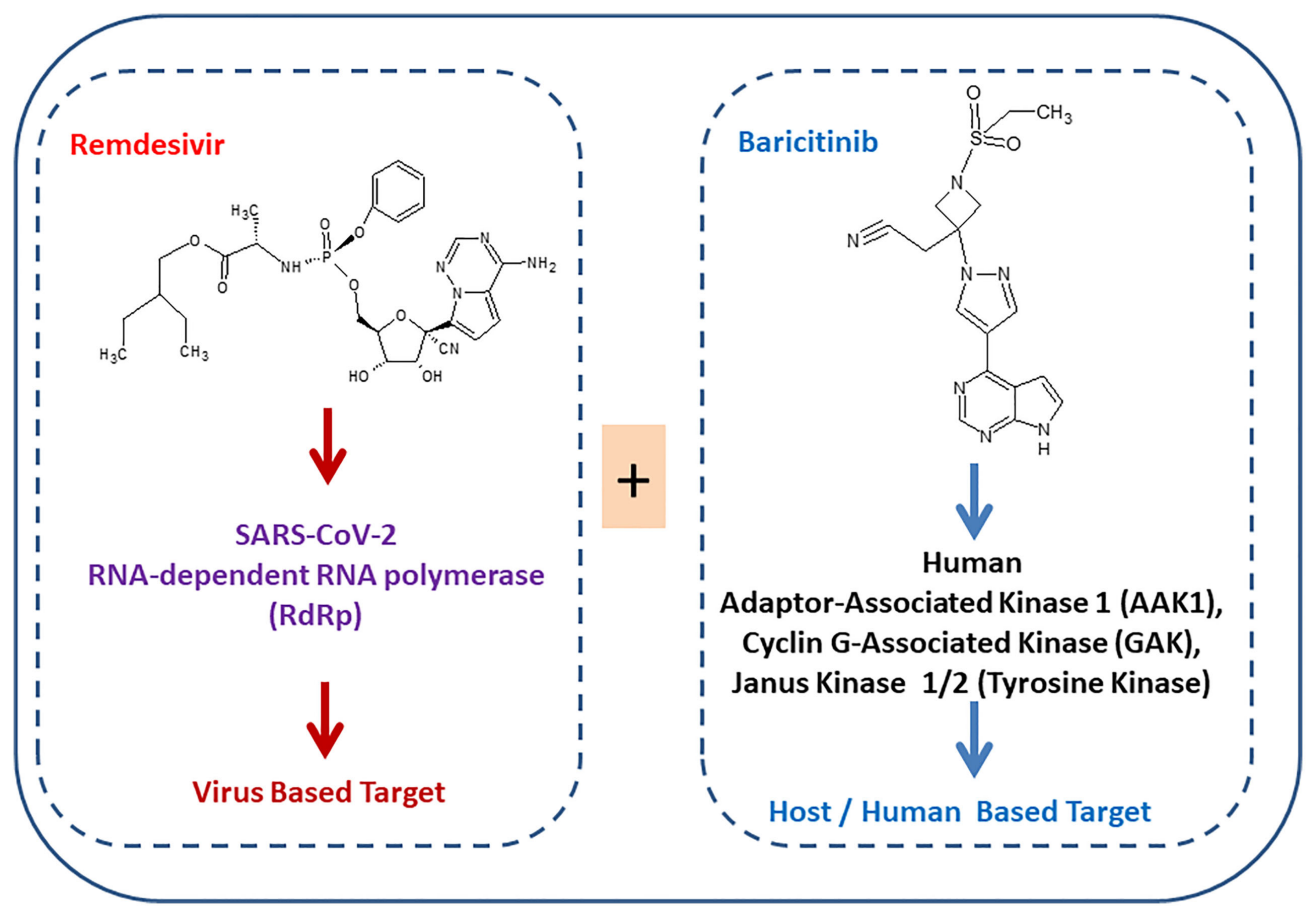
Simultaneous targeting of virus enzyme by remdesivir and human kinase by baricitinib.

Several drugs are in clinical trials with remdesivir as combinatorial therapy for COVID-19 (**Figure 3**) (42). Notably, US-FDA has also approved the remdesivir–baricitinib combination as an emergency drug for the treatment of COVID-19 (**Figure 4**) (43). Remdesivir inhibits viral RdRp while baricitinib inhibits host human multiple kinases such as JAK 1/2, AAK1, and cyclin GAK (**Figure 5**). Interestingly, the crystal structure of the host Human JAK2 JH1 with baricitinib has been deciphered at 1.9 Å using x-ray diffraction (**Figure 2**) (44, 45).

We provided a review of the approved and potential uses of remdesivir and its combinations against SARS-CoV-2, with emphasis on the combination of repurposed drugs that modulate viral and host targets.

## Mode of Action of Remdesivir in SARS-CoV-2 Infection

After entering the cells, remdesivir is metabolized into alanine metabolite (GS-704277) (**Figures 1**, **2**). In the presence of phosphoramidase-type enzymes, it is converted to nucleoside monophosphate and finally to nucleoside triphosphate, which inhibits RdRp and the replication of the virus (**Figure 1**) by inducing delayed chain termination (46). RdRp successfully inserts the active triphosphate (from remdesivir) into RNA at position i, thereby causing termination of the RNA synthesis at the i3 position (47). Similar results were also reported for SARS-CoV, MERS-CoV, and SARS-CoV-2.

### Mechanism of Remdesivir-Induced RdRp Stalling in SARS-CoV-2

Remdesivir is used as an emergency drug in an ongoing pandemic caused by SARS-CoV-2. The active form of remdesivir inhibits RdRp of SARS-CoV-2. Furthermore, remdesivir is integrated by RdRp into the increasing RNA product and allows the inclusion of three more nucleotides before the RNA synthesis stalls. Kokic et al. have attempted to understand the molecular mechanism of remdesivir-induced RdRp stalling. They found that the inclusion of the fourth nucleotide after remdesivir incorporation into the RNA product was disturbed by the RNA translocation barrier. The RNA translocation barrier causes preservation of the RNA 3′-nucleotide in RdRp and intervenes in the next nucleoside triphosphate entry causing RdRp stalling (38, 48).

### Mechanism for Remdesivir Action on the RNA Template-Dependent Inhibition of RdRp

Remdesivir may inhibit the RNA synthesis using SARS-CoV-2 RdRp by both incorporating in the primer strand and also using the template strand. Furthermore, both modes of inhibition may be connected to interactions between the conserved residues in the nsp12 and 1’-CN group of the nucleotide analog. The side chain of Ser-861 was responsible for steric clash with the active form of remdesivir, and inhibition was observed at position i+13. The backbone of Ala-558 was responsible for steric problems during the incorporation of uridine triphosphate (UTP) opposite remdesivir in the template, and inhibition was observed at positions i and i+11. Furthermore, the V557L resistance-associated mutation tries to counteract this effect. The template-dependent inhibition of SARS-CoV-2 RdRp by remdesivir has a biological significance based on resistance-conferring mutation and template-dependent inhibition (49).

The dominant mechanism for remdesivir action is difficult to find. The delayed chain termination mechanism is related to low concentrations of nucleoside triphosphate (NTP). Efficient use of the active triphosphate form of remdesivir may lead to several incorporation events such as polyU-tracts that may amplify inhibition. Interestingly, template-dependent inhibition mechanisms can be observed at higher NTP concentrations and depend on some protection from proofreading that may retain remdesivir in copies of the viral genome (49). Notably, the above mechanisms of remdesivir action may help search for several better compounds to inhibit SARS-CoV-2 replications.

## Antiviral Effect of Remdesivir

### Pre-Clinical Studies of COVID-19 Treated With Remdesivir

Remdesivir inhibits SARS-CoV-2 infection at a low micromolar concentration of 0.77 µM with EC_50_ at 48 h in Vero E6 cells infected with the SARS-CoV-2 virus (50). Another study proved that remdesivir inhibits the replication of SARS-CoV-2 in Calu3 human lung cells with sub-micromolar effective concentration (EC_50_) and with nanomolar EC_50_ in cultures of primary human airway epithelial (HAE) cells. Furthermore, remdesivir inhibits chimeric SARS-CoV expressing SARS-CoV-2 RdRp in mice by improving its pulmonary function (51). Remdesivir has been seen to be effective after 12 h of treatment in a SARS-CoV-2-infected monkey (rhesus macaque), where there were no symptoms of respiratory disease, and the virus titers have also been reduced in bronchoalveolar lavages (52). Notably, viral shedding did not decrease from the upper respiratory tract, but the damage to the lungs was reduced.

### Clinical Studies of COVID-19 Treated With Remdesivir

The promising result of remdesivir treatment was seen in the first clinical case of COVID-19 in the US (53). Later, the trial was carried out to find the efficacy and safety of remdesivir treatment in hospitalized COVID-19 patients (54). Remdesivir is given intravenously with 200 mg on the first day followed by 100 mg for 9 days in adults. In pediatric patients, the dose was adjusted according to body weight (46).

The compassionate use of remdesivir was provided to COVID-19 patients with an oxygen saturation of 94% or less in a cohort study. The patients were administered 200 mg of remdesivir intravenously on the first day, and then the dose was reduced to 100 mg and was continued for the remaining 9 days in a 10-day course of remdesivir. Clinical improvement including in oxygen support was seen in 36 out of 53 (about 68%) hospitalized patients with severe COVID-19 (55). Another compassionate study of a 10-day course of remdesivir was carried out in COVID-19 patients with pneumonia, aged >18 years old, with mechanical ventilation or an oxygen level of <94%, who were hospitalized in an intensive care unit (ICU) and a non-ICU. The study suggested that the non-ICU patients responded well to remdesivir, with better clinical outcomes and fewer adverse effects, but ongoing trials will clear its genuine efficacy and safety (56).

There was not much difference between the 5-day and 10-day course of remdesivir for patients with severe COVID-19 that did not need mechanical ventilation (57). A clinical phase 3, randomized, double-blind, placebo-controlled trial was studied, with 200 mg of remdesivir administered intravenously on the first day, followed by 100 mg remdesivir daily for 9 days and placebo for 10 days continuously to COVID-19 adult patients with lower respiratory tract infection. Preliminary results showed that there was an improvement in the duration of recovery in 538 COVID-19 patients administered with remdesivir compared to 521 patients given the placebo. Notably, patients treated with remdesivir recovered in 11 days while those who received the placebo recovered in 15 days; thus, an improvement in recovery time of 4 days was observed (58). A comparative study was carried out with remdesivir versus standard-of-care treatment without remdesivir for patients with severe COVID-19, and it was found that remdesivir was associated with lower mortality and rising recovery rate (59). It was also found that remdesivir only suppresses SARS-CoV-2 infection in immunocompromised patients diagnosed with chronic lymphocytic leukemia (60); however, in pediatric acute lymphoblastic leukemia with accompanying SARS-CoV-2 infection, 5 mg/kg of remdesivir given intravenously, followed by a dose of 2.5 mg/kg of remdesivir daily resulted in the child being SARS-CoV-2 negative (61). Notably, late initiation of remdesivir also worked well in the treatment of SARS-CoV-2-infected persons when it was administered on the 13th day of SARS-CoV-2 infection (62).

The promising result from the intravenously administered remdesivir in COVID-19 patients prompted a clinical trial where remdesivir, in a nebulized formulation, is delivered *via* inhalation to the upper respiratory tract, which is the primary site of SARS-CoV-2 infection. This is the targeted administration of the drug and would be helpful for non-hospitalized patients along with less systemic exposures to the drug. Gilead Sciences announced the phase 1 clinical study with inhalable remdesivir for the treatment of COVID-19 (63). Sun (64) suggested the evaluation of the combination of nebulizer inhalation and intravenous (IV) administration of remdesivir against COVID-19. Furthermore, it was suggested that if COVID-19 becomes a seasonal disease, then a mid-term strategy with the combination of dry powder inhalation and IV administration of remdesivir should be investigated.

## Antiviral Effect of Remdesivir Derivatives

Nucleoside analogs belong to an important group of antivirals that interfere with the highly conserved active center of viral polymerases. Remdesivir, a nucleoside analog, is the first approved anti-SARS-CoV-2 drug, although it has its limitations including less therapeutic efficacy for severe COVID-19 cases (65). Furthermore, two other oral nucleoside analogs entered phase II/III clinical studies, namely, molnupiravir (EIDD-2801) and AT-527 (66, 67). Xie et al. have screened several nucleoside/nucleotide analogs for anti-SARS-CoV-2 activity in Vero E6 cells that were known as antiviral agents and had inhibitory activities against one or multiple viruses while remdesivir (EC_50_ = 1.71 μM) and its parent nucleoside GS-441524 (EC_50_ = 0.59 μM) significantly inhibited the replication of SARS-CoV-2 (68).

The oral remdesivir derivative, VV116, was administered in hACE2-transduced mice to determine its anti-SARS-CoV-2 efficacy with positive control-EIDD-2801. Xie et al. have reported VV116 as a safe and oral anti-SARS-CoV-2 nucleoside that has significant drug-like properties. VV116 is a modified structure of GS-441524. Furthermore, the nucleoside triphosphate form of VV116 targets viral RNA-dependent RNA polymerase with an IC_50_ of 0.67 μM. Pre-clinical studies suggest that VV116 is a significantly effective oral nucleoside drug against SARS-CoV-2 (68).

Lo et al. reported an oral ODBG-P-RVn (lipid-modified monophosphate prodrug of the remdesivir parent nucleoside GS-441524), which showed a 20-fold higher antiviral activity than GS-441524 and had an activity very similar to that of remdesivir using primary-like human small airway epithelial cells (69).

Cox et al. reported that oral administration of 10 mg/kg GS-621763 (oral prodrug of remdesivir-related nucleoside GS-441524) twice daily almost completely inhibits SARS-CoV-2 in ferrets. Furthermore, oral GS-621763 is successfully converted to plasma metabolite GS-441524 and further in the lungs to triphosphate metabolite in a similar fashion to that generated by remdesivir. Notably, GS-621763 therapeutics against VOC P.1 gamma prevents viral transmission and blocks virus replication in untreated contact animals. The direct administration of GS-441524 has a less efficient metabolism compared to its monophosphate; therefore, to generate active triphosphate GS-443902 in lung tissues, there is a need for higher daily systemic exposures of GS-441524 compared to those obtained from IV remdesivir (70).

## Bioavailability and Biosafety of Remdesivir and Its Derivatives

Remdesivir is administered only by IV infusion as it has suboptimal oral bioavailability; therefore, prophylactic use of remdesivir is also hampered. Remdesivir showed low bioavailability using pharmacokinetic experiments in cynomolgus monkeys. The IV administration of 10 mg/kg remdesivir is quickly converted to nucleoside phosphate in rhesus monkeys. Furthermore, remdesivir quickly spread in peripheral blood mononuclear cells (PBMCs) within 2 h and is further activated to nucleoside triphosphate and has a survival rate of 100% (71, 72). Remdesivir was administered once a day, and its duration of action is moderate. Remdesivir was absorbed quickly and maximal plasma concentrations were reached in 0.67–0.68 h (*T*_max_) after a single 30-min IV dose while a *C*_max_ (coefficient of variation as a percentage) of 2,229 (19.2) ng/ml was observed after the repetitive dose. Furthermore, remdesivir metabolite GS-441524 had a *T*_max_ of 1.51–2.00 h and a *C*_max_ of 145 (19.3) ng/ml, while GS-704277 had a *T*_max_ of 0.75 h and a *C*_max_ of 246 (33.9) ng/ml. Remdesivir has a very short life, as after IV infusion, the peak serum concentrations were measured to be about 3 mg L^−1^, while after 1 h, it declined to 80–170 μg L^−1^ (73, 74).

There is a requirement for orally bioavailable antiviral drugs for early therapeutics of SARS-CoV-2 infection and several compounds are under clinical development including molnupiravir and PF-07321332. Notably, an active nucleoside analog against SARS-CoV-2 called EIDD-1931 is orally bioavailable (13, 75). Schooley et al. attempted to make orally bioavailable lipid analogs of remdesivir nucleoside (RVn; GS-441524) that were further processed as RVn monophosphate by a single-step intracellular cleavage and later became active RVn triphosphate. Oral lipid prodrugs of RVn have high oral bioavailability, stability in plasma, simpler metabolic activation, and submicromolar anti-SARS-CoV-2 activity in various cells including Vero E6, Caco-2, Calu-3, human pluripotent stem cell (PSC)-derived lung cells, and Huh7.5 cells (76). Oral treatment with ODBG-P-RVn (1-O-octadecyl-2-O-benzyl-glycero-3-phosphate RVn) was well tolerated in Syrian hamsters and showed better effective concentration as the therapeutic levels in plasma were observed above 90% effective concentration (EC_90_) for SARS-CoV-2 (76). GS-441524 showed poor oral bioavailability in several species while GS-621763 showed high oral bioavailability in animals (70).

Notably, GS-441524 has the potential to reach plasma concentrations up to 1000-fold higher compared to remdesivir. Fluoxetine also has high bioavailability; plasma levels of 350 µg/L were observed after oral administration of 20 mg/day for 2 weeks (77). Furthermore, the oral remdesivir derivative VV116 showed high oral bioavailability, reaching 80% in rats and 90% in dogs (68).

Interestingly, remdesivir has a favorable safety profile and is approved to treat COVID-19. It is also well-tolerated and there has been no adverse effects reported; no nephrotoxicity in healthy subjects; and no vital and clinically relevant changes and electrocardiograms (75). The IV infusions of remdesivir showed good safety and pharmacokinetic properties. Furthermore, cytotoxicity, hepatorenal toxicity, and serious adverse effects were not observed. Remdesivir with 150 mg administered intravenously daily for 7–14 days showed good tolerance, and the multi-dose study did not cause any renal injury (72). Administration of a daily dose of remdesivir (75 mg up to 225 mg IV) up to 14 days for therapeutics has no severe adverse events. Remdesivir treatment has caused minor changes such as an increase in alanine aminotransferase (ALT)/aspartate aminotransferase (AST) levels (75). Saroyo et al. reported five cases of remdesivir treatment in pregnant women with moderate to severe symptoms of COVID-19 and observed shortened hospitalization, and clinical improvement with no adverse events in mothers, fetuses, and neonates. Remdesivir showed no adverse effects in pregnant women during hospitalization and is clinically improved with shortened recovery in moderate to severe symptoms of COVID-19. Furthermore, randomized controlled trials (RCTs) are needed to evaluate the detailed biosafety of remdesivir in pregnant women (78). Shah et al. reported that the use of remdesivir treatment in five pregnant women with moderate to severe symptoms of COVID-19 resulted in a positive fetal outcome without birth defects or malformations. Furthermore, no adverse events in pregnant women, fetuses, and neonates were observed when remdesivir was administered after the period of organogenesis (first trimester) (79). However, remdesivir could cause hypersensitivity reactions, anaphylaxis, and elevated transaminase levels. Remdesivir interacts with chloroquine and hydroxychloroquine; therefore, its efficacy decreases in combination with chloroquine and hydroxychloroquine (80).

The oral remdesivir derivative VV116 is well tolerated (2.0 g/kg in rats and 1.0 g/kg in Beagle dogs) with no adverse effect after 14 days of repeated dose-toxicity studies (200 mg/kg in rats and 30 mg/kg in dogs) and showed no mutagenicity (68).

## Remdesivir Combined With Repurposed Drugs

The repurposing of drugs has become very helpful during the ongoing COVID-19 pandemic as it helps to find low-cost therapeutics in less time (77). There are more than 10 ongoing clinical trial studies on remdesivir with repurposed drugs as potential COVID-19 therapeutics (**Table 1**) (**Figure 3**). Targeting both virus enzyme and host human enzyme at the same time in COVID-19 patients using specific inhibitors has great potential to control COVID-19 as compared to targeting individually. The drugs in combination with remdesivir in ongoing clinical trials were earlier used for the treatment of different diseases. The combination treatment of host- and virus-directed drugs may overcome toxicity and resistance problems and also enhance the therapeutics for SARS-CoV-2 (74). Combination therapies are advantageous over monotherapies due to less toxicity, more efficacy, non-resistant viral strains, and the ability to handle viral co-infections.

**Table 1:** Remdesivir in combination with repurposed drugs as potential COVID-19 therapeutics.

Sl. No.	Drug Name	Title of the Clinical Trial	Clinical Trial No./Status
1	Remdesivir + Tocilizumab	Efficacy and Safety of Remdesivir and Tocilizumab for the Management of Severe COVID-19: A Randomized Controlled Trial	NCT04678739 Recruiting
2	Remdesivir + Interferon beta-1b	IFN-beta 1b and Remdesivir for COVID-19	NCT04647695 Recruiting
3	Remdesivir + Lopinavir/Ritonavir	Comparison of Remdesivir Versus Lopinavir/Ritonavir and Remdesivir Combination in COVID-19 Patients	NCT04738045 Recruiting
4	Remdesivir + Merimepodib	Study of Merimepodib in Combination With Remdesivir in Adult Patients With Advanced COVID-19	NCT04410354 Terminated
5	Remdesivir + DWJ1248	Efficacy and Safety of DWJ1248 With Remdesivir in Severe COVID-19 Patients	NCT04713176 Recruiting
6	Drug: Remdesivir + Hydroxychloroquine + Tocilizumab	Comparison of Remdesivir and Tocilizumab Versus Hydroxychloroquine and Tocilizumab Combination in COVID-19 Patients	NCT04779047 Recruiting
7	Remdesivir + Tocilizumab	A Study to Evaluate the Efficacy and Safety of Remdesivir Plus Tocilizumab Compared With Remdesivir Plus Placebo in Hospitalized Participants With Severe COVID-19 Pneumonia	NCT04409262 Active, not recruiting
8	Remdesivir + Interferon beta-1a	Treatments for COVID-19: Canadian Arm of the SOLIDARITY Trial	NCT04330690 Recruiting
9	Remdesivir + Baricitinib	Adaptive COVID-19 Treatment Trial 2 (ACTT-2)	NCT04401579 Completed
10	Remdesivir + Interferon beta-1a	Adaptive COVID-19 Treatment Trial 3 (ACTT-3)	NCT04492475 Completed
11	Remdesivir + Baricitinib + Tocilizumab	Efficacy of Remdesivir and Baricitinib for the Treatment of Severe COVID-19 Patients	NCT04693026 Recruiting
12	Remdesivir + Lenzilumab	ACTIV-5/Big Effect Trial (BET-B) for the Treatment of COVID-19	NCT04583969 Recruiting
13	Remdesivir + Risankizumab	ACTIV-5/Big Effect Trial (BET-A) for the Treatment of COVID-19	NCT04583956 Recruiting

### Combination of Remdesivir With Baricitinib

US-FDA has approved a combination of baricitinib–remdesivir as an emergency drug for the treatment of COVID-19 on November 19, 2020. Notably, remdesivir targets virus enzymes while baricitinib targets host human enzymes simultaneously in COVID-19 patients. Baricitinib is a reversible and selective inhibitor of Janus kinase belonging to the family of tyrosine protein kinase. Notably, baricitinib has also been predicted as a potential drug candidate for SARS-CoV-2 using artificial intelligence (AI) algorithms (26, 27). Furthermore, the Adaptive COVID-19 Treatment Trial (ACTT-2) as a combination treatment with baricitinib and remdesivir was better than remdesivir treatment, especially in reducing the recovery time in patients receiving high-flow oxygen or noninvasive mechanical ventilation. Moreover, baricitinib–remdesivir-treated patients showed a higher likelihood of improved clinical status at day 15 compared to the group who received only remdesivir (28). Kalil et al. have reported that baricitinib–remdesivir has fewer adverse effects (28).

Kalil et al. carried out a double-blind, randomized, placebo-controlled trial to evaluate the combination of baricitinib plus remdesivir in hospitalized COVID-19 adult patients by assigning 515 patients to the combination of remdesivir and baricitinib. Remdesivir was given for ≤10 days and baricitinib was administered for ≤14 days. Furthermore, 518 patients that were considered as control received remdesivir for ≤10 days with placebo (Clinical Trial No. NCT04401579). The patients who received a combination of remdesivir and baricitinib recovered in a median time of 7 days compared to the control group with a recovery time of 8 days. The patients who received a combination of remdesivir and baricitinib at day 15 had 30% higher odds of improvement in their clinical status. Furthermore, for the patients requiring high-flow oxygen or noninvasive ventilation, the recovery for those who received a combination of remdesivir and baricitinib occurred in 10 days compared to the recovery of 18 days for the control group. Notably, the 28-day mortality was 5.1% in the patients who received a combination of remdesivir and baricitinib compared to 7.8% in the control group. The serious adverse events were less observed in patients who received a combination of remdesivir and baricitinib compared to the control group. Importantly, the combination of remdesivir and baricitinib gave fewer serious adverse effects (28).

Interestingly, the majority of ongoing clinical trials use remdesivir in combination with baricitinib/tocilizumab/DWJ1248/risankizumab/interferon beta-1a/interferon beta-1b/merimepodib/lenzilumab (**Table 1**). Moreover, some ongoing clinical trials use remdesivir in combination with two other drugs such as baricitinib–tocilizumab, hydroxychloroquine–tocilizumab, and lopinavir–ritonavir (**Table 1**).

### Combination of Remdesivir With Direct Antiviral Agents or Host-Directed Therapy

Remdesivir in combination with methotrexate (MTX) led to synergistic impairment of the replication of SARS-CoV-2 as MTX improves remdesivir efficacy in treated Vero cells by reducing the RNA virus in the cell supernatant by more than 98% while each drug alone reduces the RNA virus by 50%–75% (81).

Interestingly, remdesivir with recombinant soluble ACE2 in high/low doses as combination therapy was found to target SARS-CoV-2 entry through ACE2 receptor and intracellular viral RNA replication in kidney organoids and Vero E6, and this works well at sub-toxic concentrations. Furthermore, adenylate kinase 2 is an important pathway for remdesivir toxicity (82). Notably, a combination of 200 μg/ml of human recombinant soluble (hrs) ACE2 and 4 μM of remdesivir reduced 60% of the viral load than hrs ACE2 alone in Vero E6 cells. Interestingly, a *C*_max_ of about 7.3 μM was observed after a 225-mg dose of remdesivir for healthy volunteers (83), which works well with pharmacokinetics (PK) and loading doses for critically ill COVID-19 patients (73). Furthermore, 5 and 10 μg/ml low doses of hrs ACE2 and a low dose of remdesivir achieved additive effects and caused a significant reduction of SARS-CoV-2 infectivity in both Vero E6 cells and kidney organoids. Moreover, about 5–10 μg/ml of hrs ACE2 was observed in plasma after 2 to 8 h of administration of 800 μg/kg. Treatment with a low dose of hrs ACE2 caused a significant reduction in the yield of progeny virus at both 15 and 48 hpi, with the latter working better. Furthermore, a low dose of remdesivir had a minor effect on the yield of progeny virus at both time points. Intriguingly, both remdesivir and hrs ACE2 together caused a significant reduction in the yield of progeny virus by inhibition of viral entry and replication of SARSCoV-2. In addition, ACE2 works as a negative regulator for the renin–angiotensin system (RAS) in several tissues, including the cardiovascular system, and ACE2 protects the lung from injury, failure, and death in SARS-CoV infections (82).

The combination therapy of antiviral remdesivir metabolite GS-441524 and host-targeted antidepressant fluoxetine was well tolerated and showed synergistic antiviral effects against SARS-CoV-2 and its variants alpha and beta *in vitro* using a polarized Calu-3 cell culture model. Therefore, this combination therapy was also effective in SARS-CoV-2 variants with mutations in spike proteins. Notably, the remdesivir metabolite GS-441524 had EC_50_ (0.28 µM) and EC_90_ values (1.33 µM) in polarized Calu-3 cells comparable with remdesivir, which had an EC_50_ of 0.28 µM and an EC_90_ of 2.48 µM (77). The combination therapy of GS-441524 with fluoxetine had synergistic activity comparable to that of the combination of remdesivir with fluoxetine. Furthermore, the monotherapy of GS-441524 and fluoxetine needs a higher dose while the combination therapy of GS-441524 with fluoxetine provides a synergistic action and needs a lower concentration of individual drugs that reduce side effects; therefore, no cytotoxic effect is observed.

Schloer et al. reported that the combination of itraconazole–remdesivir and fluoxetine–remdesivir showed synergistic effects and inhibited more than 90% of SARS-CoV-2 particle production. The antifungal–itraconazole and antidepressant–fluoxetine combination with viral RNA polymerase inhibitor–remdesivir showed better antiviral potency through synergy *in vitro* in Calu-3 cells (74).

Sheahan et al. reported that remdesivir (EC_50_: 0.09 µM) and interferon beta (IFNb) (EC_50_: 175 IU/ml) had better antiviral activity against the Middle East respiratory syndrome coronavirus (MERS-CoV) compared to lopinavir (EC_50_: 11.6 µM) and ritonavir (EC_50_: 24.9 µM) *in vitro*. Furthermore, remdesivir as a prophylactic and therapeutic was able to reduce lung viral loads and severe lung pathology and also improved the lung function in mice against MERS-CoV (84). Furthermore, Ianevski et al. reported that interferons (IFNs) in combination with remdesivir were effective against SARS-CoV-2 while interferons alone were unable to eliminate the replication of SARS-CoV-2. The combination of IFNα2a–remdesivir vanquished the SARS-CoV-2 effect in both Calu-3 cells and lung organoids while a change in the homeostasis of uninfected cells and organoids was observed (85).

## Clinical Potential and Limitations of Remdesivir and Its Derivatives (Alone or in Combination With Other Drugs)

Remdesivir (RDV; GS-5734) is the first emergency drug approved by the FDA for the treatment of COVID-19 (74). However, it is unable to reduce mortality. Furthermore, its hepatic and kidney toxicities cause a hindrance in the dose of remdesivir (82). Moreover, the bioactivation of remdesivir depends on the pro-drug activating enzymes. Remdesivir is also approved for hospitalized COVID-19 patients, 12 years old and above. Remdesivir is required to be administered intravenously and is also found to be unstable in plasma and has a highly variable antiviral efficacy in SARS-CoV-2-infected cells because of the complex activation pathway (76).

GS-441524, a plasma metabolite of remdesivir, may be activated by several kinases such as adenosine kinase (ADK). Furthermore, ADK is expressed in all tissues. The pharmacokinetics of the antiviral drug GS-441524 suggests therapeutic potential against SARS-CoV-2 infection (77). Notably, GS-441524 is one of the remdesivir metabolites that have a better half-life in plasma, which significantly inhibits SARS-CoV-2 replication *in vitro* and also inhibits SARS-CoV-2 infection and pathogenesis in a mouse model (74). The oral remdesivir derivative VV116 showed dose-dependent efficacy with lowering copies of viral (SARS-CoV-2) RNA and titers of the infectious virus (SARS-CoV-2) in the lungs (68).

Schloer et al. reported that the ItraRem and FluoRem drugs showed better antiviral activities using lower concentrations against SARS-CoV-2 compared to the remdesivir monotherapy. Furthermore, plasma concentrations of these drugs were observed to be within range, and drug synergistic effects were better than the sum of the independent drug effects (74). Remdesivir with baricitinib improved the treatment of hospitalized COVID-19 patients (28).

Some side effects of remdesivir are nausea, vomiting, shivering, sweating, and having a lightheaded feeling. Remdesivir should be taken carefully with other medicines particularly when the patient has an allergy or kidney, liver, and other serious illnesses. In addition, care should be taken in prescribing P-glycoprotein (P-gp) inhibitors for COVID-19 patients having acute hepatotoxicity after receiving remdesivir treatment as remdesivir probably interacts with P-glycoprotein (P-gp) inhibitors (86).

The combination of drugs might be administered to patients after careful evaluation of their physiological, genetic disposition, or pathophysiological conditions to avoid patient-related risks and contraindications in certain patients (74). The combination therapy has a potential risk of drug–drug interaction that may sometimes cause a decrease in therapeutic benefit and cause adverse effects; therefore, it needs proper evaluation and careful therapeutics strategy for SARS-CoV-2 (77). The application of a combination strategy of the antiviral remdesivir with host-directed drugs has the potential to be successful, although there is some concern regarding the translation of these *in vivo* and *in vitro* results in clinical success such as the interaction of these drugs. There are several drugs that might interact with remdesivir, itraconazole, and fluoxetine (87). This might reduce their effect or may cause some adverse effects; therefore, it needs to be clinically verified. Notably, the safety profile and pharmacokinetic information of these drugs are known (74).

Cytochrome P450 (CYP) enzymes (CYP2C8, CYP2D6, and CYP3A4) cause metabolization of remdesivir and GS-4412524. The antidepressant fluoxetine is an inhibitor of CYPs (CYP2D6 and CYP2C9/10) and also a serotonin-reuptake inhibitor (SRI). The administration of fluoxetine needs to be monitored carefully and avoided with other SRIs including amphetamines and sympathomimetic appetite suppressants (77).

## Conclusions

The combination of remdesivir–baricitinib was used as therapeutics during the ongoing COVID-19 pandemic. Based on the available clinical and experimental data, remdesivir–baricitinib should be given to patients in the early stage of SARS-CoV-2 infection. This helps in fast recovery, stops further progression of SARS-CoV-2 infection, and controls mortality in hospitalized patients. This combination of drugs is helpful in moderate and severe SARS-CoV-2 infection with or without ventilation. However, it is not adequately effective in the case of severely sick COVID-19 patients. Therefore, the remdesivir–baricitinib combination at a very low dose can also be used as a prophylactic in suspected cases including asymptomatic and pre-symptomatic cases of COVID-19.

The remdesivir–baricitinib combination encouraged combinatorial drug strategies, thereby opening an important avenue in COVID-19 therapeutics. The combination of repurposed drugs with remdesivir may reduce the adverse effects, recovery time, and hospitalization stay and improve patient clinical status for noninvasive ventilation. Several repurposed drugs including human monoclonal antibodies and immunomodulators are under clinical trials in combination with remdesivir for the treatment of SARS-CoV-2 infection. In the future, remdesivir in combination with other certified drugs may be repurposed as SARS-CoV-2 therapeutics by simultaneously targeting virus replication enzymes and host human kinases.

Drugs targeting virus structures may eliminate the virus in a shorter treatment time before it develops drug resistance. Resistance of viruses to drugs was observed with oseltamivir, an influenza neuraminidase inhibitor (88, 89). Furthermore, for the survival of the virus, it needs to replicate and thus requires essential host factors; therefore, combining both host- and virus-directed drugs may improve viral therapeutics and overcome drug resistance problems (74).

## Author Contributors

BC and ST have conceived the idea and wrote the manuscript.

## Conflict of Interest

The authors declare that the research was conducted in the absence of any commercial or financial relationships that could be construed as a potential conflict of interest.

## Publisher’s Note

All claims expressed in this article are solely those of the authors and do not necessarily represent those of their affiliated organizations, or those of the publisher, the editors and the reviewers. Any product that may be evaluated in this article, or claim that may be made by its manufacturer, is not guaranteed or endorsed by the publisher.
